# Urinary Metabolite Profiles in Premature Infants Show Early Postnatal Metabolic Adaptation and Maturation

**DOI:** 10.3390/nu6051913

**Published:** 2014-05-12

**Authors:** Sissel J. Moltu, Daniel Sachse, Elin W. Blakstad, Kenneth Strømmen, Britt Nakstad, Astrid N. Almaas, Ane C. Westerberg, Arild Rønnestad, Kristin Brække, Marit B. Veierød, Per O. Iversen, Frode Rise, Jens P. Berg, Christian A. Drevon

**Affiliations:** 1 Department of Pediatrics, Oslo University Hospital, P.O. Box 4950 Nydalen, Oslo 0424, Norway; E-Mail: sissel.moltu@medisin.uio.no; 2 Department of Nutrition, University of Oslo, P.O. Box 1046 Blindern, Oslo 0317, Norway; E-Mails: kestro@ous-hf.no (K.S.); a.c.westerberg@medisin.uio.no (A.C.W.); m.b.veierod@medisin.uio.no (M.B.V.); p.o.iversen@medisin.uio.no (P.O.I.); c.a.drevon@medisin.uio.no (C.A.D.); 3 Department of Medical Biochemistry, University of Oslo, P.O.Box 4950 Nydalen, Oslo 0424, Norway; E-Mail: j.p.berg@medisin.uio.no; 4 Department of Medical Biochemistry, Oslo University Hospital, P.O. Box 4956 Nydalen, Oslo 0424, Norway; 5 Department of Chemistry, University of Oslo, P.O. Box 1033 Blindern, Oslo 0315, Norway; E-Mail: frode.rise@kjemi.uio.no; 6 Department of Pediatric and Adolescent Medicine, Akershus University Hospital, Lørenskog 1478, Norway; E-Mails: e.w.blakstad@medisin.uio.no (E.W.B.); britt.nakstad@medisin.uio.no (B.N.); a.n.almaas@gmail.com (A.N.A.); 7 Institute of Clinical Medicine, University of Oslo, P.O. Box 1171 Blindern, Oslo 0318, Norway; 8 Department of Neonatal Intensive Care, Oslo University Hospital, P.O. Box 4950 Nydalen, Oslo 0424, Norway; E-Mails: aronnest@ous-hf.no (A.R.); uxkbre@ous-hf.no (K.B.); 9 Atlantis Medical University College, P.O. Box 4290 Nydalen, Oslo 0402, Norway; 10 Department of Biostatistics, University of Oslo, P.O. Box 1122 Blindern, Oslo 0317, Norway; 11 Department of Hematology, Oslo University Hospital, P.O. Box 4950 Nydalen, Oslo 0424, Norway

**Keywords:** prematurity, very low birth weight, pediatric nutrition, intervention study, metabolomics, urine, nuclear magnetic resonance spectroscopy, glycine, threonine

## Abstract

Objectives: Early nutrition influences metabolic programming and long-term health. We explored the urinary metabolite profiles of 48 premature infants (birth weight < 1500 g) randomized to an enhanced or a standard diet during neonatal hospitalization. Methods: Metabolomics using nuclear magnetic resonance spectroscopy (NMR) was conducted on urine samples obtained during the first week of life and thereafter fortnightly. Results: The intervention group received significantly higher amounts of energy, protein, lipids, vitamin A, arachidonic acid and docosahexaenoic acid as compared to the control group. Enhanced nutrition did not appear to affect the urine profiles to an extent exceeding individual variation. However, in all infants the glucogenic amino acids glycine, threonine, hydroxyproline and tyrosine increased substantially during the early postnatal period, along with metabolites of the tricarboxylic acid cycle (succinate, oxoglutarate, fumarate and citrate). The metabolite changes correlated with postmenstrual age. Moreover, we observed elevated threonine and glycine levels in first-week urine samples of the small for gestational age (SGA; birth weight < 10th percentile for gestational age) as compared to the appropriate for gestational age infants. Conclusion: This first nutri-metabolomics study in premature infants demonstrates that the physiological adaptation during the fetal-postnatal transition as well as maturation influences metabolism during the breastfeeding period. Elevated glycine and threonine levels were found in the first week urine samples of the SGA infants and emerged as potential biomarkers of an altered metabolic phenotype.

## 1. Introduction

Despite improved perinatal medical care and increased focus on enhanced nutritional support to premature infants, pre- and postnatal growth-restriction still occurs in 60%–100% of infants with a very low birth weight (<1500 g) [1,2]. Premature infants with growth restriction are at risk of impaired cognitive function and adverse metabolic and cardiovascular outcomes later in life [3,4]. Metabolic changes occurring *in utero*, during birth and the postnatal weaning period, seem to be of particular importance for future health [5,6,7]. Nutritional alterations during these periods are associated with a predisposition to obesity, cardiovascular diseases and associated co-morbidities later in life [5,8]. However, the time frame for these programming effects on long-term disease risk is controversial. Present evidence favors proactive nutritional support in premature infants to promote growth similar to the intrauterine growth rate and to support cognitive development [1,3,4]. This is in contrast with the potentially advantageous effects of relative undernutrition and slower growth on long-term cardiovascular health [8].

Recently, we published results from a randomized, controlled trial comparing the effect of enhanced nutritional supply (intervention) as opposed to a standard (control) diet, on postnatal growth in premature infants with a birth weight < 1500 g [9,10]. The infants in the intervention group, with median nutrient supplies in the upper range of current recommendations (Table 1) [9,11,12], exhibited postnatal growth along their birth percentiles for both weight and head circumference, whereas the control infants fell off their expected growth trajectories from birth to 36 weeks postmenstrual age (PMA). However, a preplanned safety analysis after the enrolment of 50 infants revealed an increased occurrence of late onset septicemia without increased mortality in the intervention group, and it was decided to halt further recruitment [10].

**Table 1:** Daily nutrient supply up to four weeks after birth.

	Intervention (*n* = 23)	Control (*n* = 21)	*p*
Human milk, mL/kg/day	133 (110–139)	134 (124–141)	0.37
Energy, kcal/kg/day	139 (128–145)	126 (121–128)	<0.001
Protein, g/kg/day	4.0 (3.9–4.2)	3.2 (3.1–3.3)	<0.001
Lipids, g/kg/day	7.3 (6.4–7.6)	5.9 (5.6–6.1)	<0.001
Carbohydrates, g/kg/day	14.4 (13.4–14.8)	14.7 (14.3–15.1)	0.12
Arachidonic acid, mg/kg/day	68 (57–73)	24 (23–25)	<0.001
Docosahexaenoic acid, mg/kg/day	87 (81–91)	36 (34–38)	<0.001
Vitamin A, μg/kg/day	1300 (1105–1442)	252 (238–257)	<0.001

Detailed records of actual nutrient supply was available for 44 infants [9]. Data are presented as medians (interquartile ranges) and compared using the Mann-Whitney U test.

To assess the metabolic status of these premature infants and to explore potentially different responses to the two diets, we used state-of-the-art nuclear magnetic resonance (NMR)-based metabolomics to analyze urine samples obtained during the postnatal period. Metabolomics is recognized as a powerful top-down systems biology approach that explores the genetic-environment-health interaction [13,14]. The approach is to obtain broad snapshots of the metabolism by detecting and quantifying hundreds of small-molecular substances (molecular mass < 1000 Da) in tissues or body fluids, and then link them to disease or development states using multivariate statistical methods such as principal component analysis (PCA) and partial least squares (PLS) regression that handle and integrate large datasets [15,16]. Metabolomics of biofluids is thought to be a promising new tool in neonatology, especially in premature infants, due to its comprehensive and usually non-invasive nature [17]. Metabolomic analysis of urine from the neonatal period may be used to understand metabolic processes linked to early nutrition. It may also be used to identify biomarkers for diagnosis, prognosis and risk prediction of different diseases [5,13,17,18,19].

The main objectives of the present study were to analyze urine samples from the premature infants of the previous trial in relation to the two different nutritional exposures and to assess the infants’ postnatal metabolic maturation. Secondary objectives were to explore potential differences related to age, sex, infections as well as pre- and postnatal growth.

## 2. Materials and Methods

### 2.1. Study Design and Population

The study was part of an open, randomized, controlled clinical trial [9,10], approved by the Regional Committee for Medical and Health Research Ethics and in accordance with the principles of the Helsinki Declaration. Fifty premature infants with birth weight < 1500 g were recruited from the neonatal intensive care units at Oslo University Hospital and Akershus University Hospital, Norway, from 17 August to 21 December 2010; 24 in the intervention group and 26 in the control group. Exclusion criteria were congenital malformations, chromosomal abnormalities, critical illnesses with short life expectancy and clinical syndromes known to affect growth and development. Morbidities were registered according to routine clinical practice and standard definitions [20,21,22,23]. Infants were classified as small for gestational age (SGA) if their birth weight was below the 10^th^ percentile of a reference population [24], or as appropriate for gestational age (AGA) otherwise. Growth velocity was calculated by the exponential equation described by Patel *et al.* [25].

Two infants in the control group died during the first week of life, leaving 48 infants for the analysis [10]. Demographic and clinical characteristics are presented in Table 2. The significantly higher occurrence of septicemia and electrolyte deviations observed in the intervention group have been reported previously [10].

**Table 2:** Baseline characteristics and clinical outcomes.

	Intervention (*n* = 24)	Control (*n* = 24)	*p*
Gestational age (weeks), mean (range)	28.1 (25.0–33.6)	28.5 (24.0–32.6)	0.60
Birth weight (g), mean (range)	940 (460–1311)	1083 (571–1414)	0.03
Small for gestational age, *n* (%)	11/24 (46%)	5/24 (21%)	0.12
Sex, boys, *n* (%)	15/24 (63%)	15/24 (63%)	1.00
Cesarean section, *n* (%)	16/24 (67%)	19/24 (79%)	0.52
APGAR-score, 5-min, mean (± SD)	7.33 (± 1.7)	7.54 (± 1.7)	0.68
Prenatal steroid treatment, *n* (%)	22/24 (92%)	24/24 (100%)	0.49
Late onset septicemia, *n* (%)	15/24 (63%)	7/24 (29%)	0.04
NEC, *n* (%)	1/24 (4%)	2/24 (8%)	1.00
IVH, grade ≥3, *n* (%)	2/24 (9%)	2/24 (9%)	1.00
PVL, grade ≥3, *n* (%)	0/24 (0%)	1/24 (4%)	1.00
ROP (severe grade III/+disease), *n* (%) ^a^	3/23 (13%)	2/23 (9%)	1.00
O_2_ at 36 weeks PMA, *n* (%) ^a^	5/23 (22%)	6/23 (26%)	1.00
PDA surgical treatment, *n* (%)	4/24 (17%)	2/24 (8%)	0.67
Deaths before 36 weeks PMA, *n* (%)	1/24 (4%)	1/24 (4%)	1.00
Hypophosphatemia 1st week, *n* (%) ^b^	17/22 (77%)	6/23 (26%)	0.001
Hypokalemia 1st week, *n* (%)	21/24 (88%)	11/24 (46%)	0.005

Student *t*-test or Fisher’s exact test was applied as appropriate. NEC: necrotizing enterocolitis; IVH: intraventricular hemorrhage; PVL: periventricular leukomalacia; ROP: retinopathy of prematurity; PDA: persistent ductus arteriosus; PMA: post-menstrual age; Hypophosphatemia < 1.4 mmol/L; Hypokalemia < 3.5 mmol/L. ^a^ Two more infants died during hospitalization, leaving 46 infants in the analyses of ROP and O_2_-dependency at 36 weeks PMA; ^b^ Only 45 infants had their phosphate concentrations measured during the first week of life.

### 2.2. Nutritional Intervention

The nutritional intervention was started on the first day of life, after informed consent was obtained [9,10]. The intervention group started with 3.5 g/kg/day of amino acids and 2.0 g/kg/day of intravenous lipids, whereas the control group started with 2.0 g/kg/day of amino acids and 0.5 g/kg/day of lipids. To improve the parenteral supply of the long chain polyunsaturated fatty acids (PUFAs), the intervention group received a lipid emulsion containing marine omega 3 fatty acids (SMOF^®^, Fresenius Kabi Norge AS, Oslo, Norway), whereas the control group received the lipid emulsion used in our units (Clinoleic^®^, Baxter AS, Oslo, Norway). The supply of human milk was increased equally in both groups, and standard fortification with 4.2 g Nutriprem^®^ (Nutricia Norge AS, Oslo, Norway)/100 mL human milk was initiated when the infants tolerated 110 mL/kg/day as enteral supply. In addition to standard fortification, the intervention group was given 0.6 g Complete Amino Acid Mix^®^ (Nutricia Norge AS, Oslo, Norway)/100 mL human milk, 60 mg/kg/day of docosahexaenoic acid (DHA; 22:6, *n*-3) as well as arachidonic acid (AA; 20:4, *n*-6), and 1500 μg/kg/day vitamin A (Ås Laboratory, Ås, Norway). On average the energy supply was approximately 10% higher and the protein supply 25% higher in the intervention group as compared to the control group [9].

### 2.3. Sample Collection and Preparation

**Figure 1:**
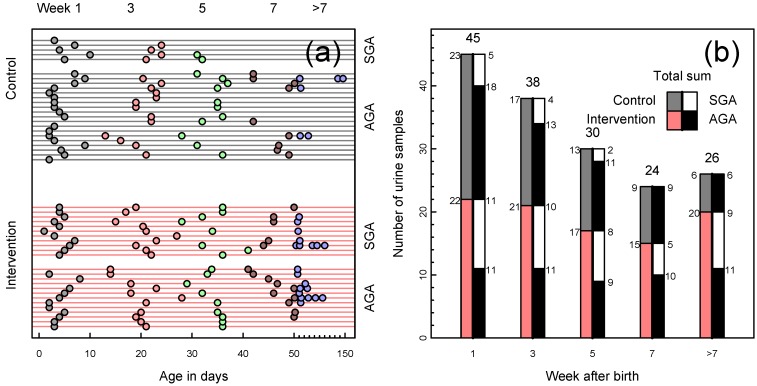
(**a**) Available urine samples by infants’ age in days, one infant per line, one sample per symbol. Grouped by intervention and control (red and gray lines, respectively), color-coded by week of life. Age in days was imputed for eight samples where only the week was recorded; (**b**) Available urine samples by infants’ week of life. Bars divided by nutritional intervention *vs.* control (left half of bar; red and gray, respectively) and further subdivided by small for gestational age (SGA) or appropriate for gestational age (AGA) infants (right half of bar; SGA white, AGA black).

Urine samples were obtained from the infants during the first week of life and thereafter approximately every other week until discharge (Figure 1). We collected 0.5–1.8 mL urine non-invasively by the use of cotton pads, transferred them to Nunc^®^ CryoTubes^®^ (Nalge Nunc International, Penfield, NY, USA) before they were stored at −80 °C. The urine samples had been thawed once prior to the metabolic NMR profiling when they were acidified for electrolyte analysis by mixing 400 μL of sample with 5 μL of 1 M HCl, resulting in a pH of approximately 3.

**Figure 2:**
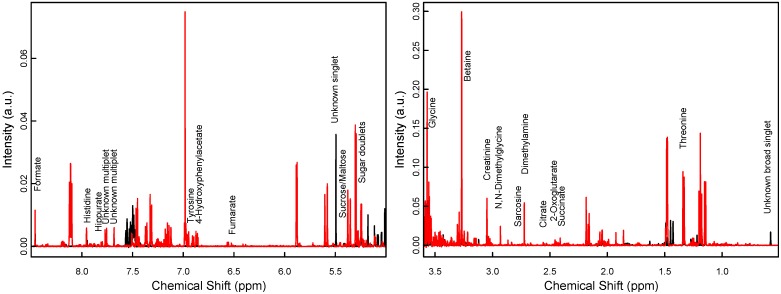
Selected regions of two NMR spectra (black for week 1 and red for week 1) of an SGA infant in the intervention group.

Metabolite profiling in the present study largely followed a protocol described earlier [26]. Briefly, 150 μL of distilled water and 50 μL of a buffer at pH 7.4 containing D_2_O and trimethylsilylpropionate-d4 (TSP) were added to 350 μL of the samples, which were then centrifuged at 13,400× *g* for 5 min and transferred to 5 mm NMR tubes (Wilmad LabGlass, Vineland, NJ, USA). One-dimensional, water-suppressed proton NMR spectra were acquired at 300.0 K on a Bruker AVI-600 spectrometer (Bruker Biospin GmbH, Rheinstetten, Germany) equipped with a TCI cryoprobe and a BACS-60 automatic sample changer, under full automation of D_2_O locking, tuning and matching, and gradient shimming using TopSpin 2.1pl6 and iconNMR. Of each sample 32 scans and 4 dummy scans were collected into 64 k data points using the Bruker “noesygppr1d.comp” sequence with a spectral width of 20.6 ppm, 2.65 s acquisition time and a 25 Hz water presaturation during the 4 s relaxation delay. An exponential line broadening of 0.3 Hz was applied. The TSP signal achieved a full width at half maximum of less than 1 Hz after apodization and acted as spectral and concentration reference. The spectra were phase-corrected, a smooth baseline was removed, and the spectra were binned to a spectral width of 0.01 ppm. Signals were assigned to known metabolites using a reference database [27] and the software Chenomx NMR Suite 7.5 professional (Chenomx Inc., Edmonton AB, Canada). Two example spectra are shown in Figure 2. Pseudo-concentrations were extracted by integrating manually defined spectral regions corresponding to both known and unknown substances, and arranged in a table. Pseudo-concentrations are proportional to absolute concentrations and can be used as such in the statistical analysis. Both the spectra and the table of metabolite pseudo-concentrations were subsequently normalized to the total intensity of the respective spectra, and the metabolite table was log-transformed.

### 2.4. Statistical Analysis

We used Student *t*-test, Mann-Whitney U test or Fisher’s exact test to evaluate differences in baseline characteristics, clinical outcomes and nutrient supplies between the two study groups [9,10]. Results are presented as frequencies (%) for categorical data, and as means (ranges or standard deviations) or medians (interquartile ranges) for continuous data [9,10]. For the metabolomics study, PCA was applied to mean-centered and unit-variance scaled spectra to explore the major variations in the dataset [28,29]. By definition, a PCA score plot arranges samples based on the similarity of their spectra, thus enabling the identification of natural groupings of and systematic changes between samples. The corresponding loadings reveal which spectral regions, i.e., which metabolites, contribute to the scores. Multivariate PLS regression was used to associate the endpoints in our study to the urine spectra. Again, the spectral variables were mean-centered and scaled to unit variance, and 7-fold cross-validation was applied to evaluate the quality of the resulting statistical models by considering the diagnostic measures R^2^ and Q^2^ [30], describing the endpoint variation captured in regression model, and the variation reproduced in cross-validation, respectively. Whereas R^2^ and Q^2^ represent measures of the strength of a multivariate relationship between profiles and endpoints, their ratio Q^2^/R^2^ is a measure of cross-validation reproducibility. In the present study, Q^2^/R^2^ ratios above 0.5 were considered indicative of relevant associations, which were then studied further.

Univariate response approaches were used on log-transformed data in the pseudo-concentration table to expand the results from the multivariate analyses. A linear mixed model for repeated measures (first-order autoregressive covariance structure) was used to study the impact of the two diets on the metabolite pseudo concentrations over time (weeks 1, 3, 5 and 7), adjusted for gestational age at birth and SGA status. Linear regression was used to quantify the relations between the metabolite pseudo concentrations at week 1 with SGA status and PMA in weeks, and also between metabolite levels, growth velocity and PMA. Results are reported as fold-change ratios (FC) with respect to back-transformed metabolite levels; ratios below 1 are presented as −1/ratio. Bonferroni correction for multiple testing was applied. The analyses were carried out on a Windows PC using SPSS version 20 (SPSS Inc., Chicago, IL, USA) and R 2.12.1, 64-bit (R Foundation, Vienna, Austria), with packages pls 2.2-0 and pcaMethods 1.32.0.

## 3. Results

**Figure 3:**
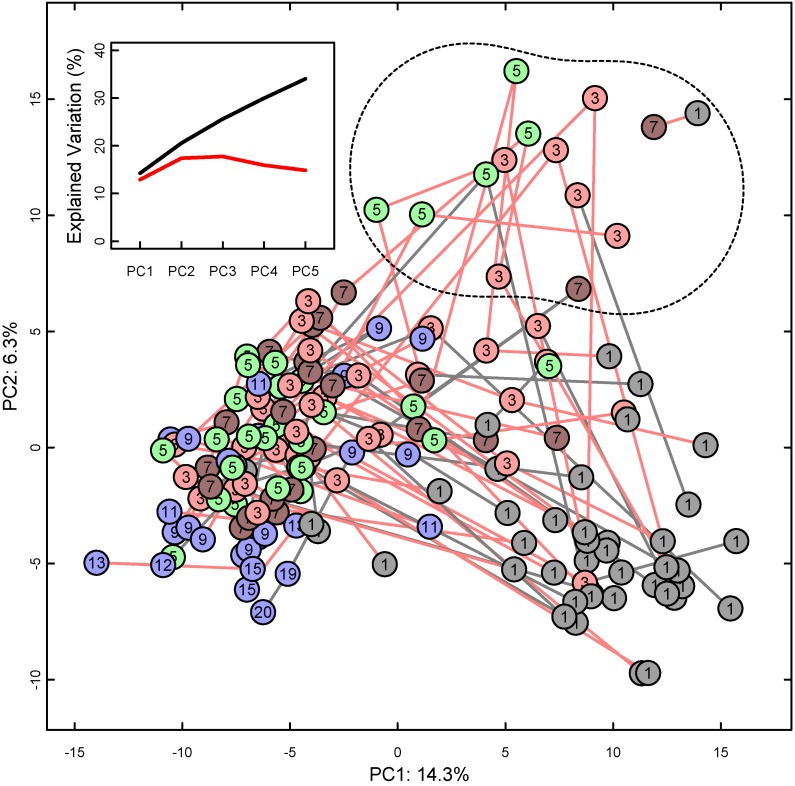
PCA score plot of NMR spectra of all available urine samples, presented as points marked with infant age in weeks and color-coded as earlier. PCA: Principal component analysis, NMR: Nuclear magnetic resonance, PC: Principal component with percent of explained total variation. Lines connect consecutive samples from one infant; line color red for intervention, gray for control group. Outlier samples marked with a dashed line in the upper right quadrant. Inset: Cumulative explained variation (black) and cross-validation (red) of the first five PCs.

The PCA score plot in Figure 3 presents the overall NMR spectroscopic relations between all the available urine samples, with lines between consecutive samples from the same infant. Urine samples from the first week of life occupy the lower right quadrant of the plot, and mostly progress towards the middle left with increasing age of the infant. There was no obvious difference in distribution or temporal development between the intervention and control group.

Several samples in the upper right quadrant deviated from this general trend and are marked as outliers. The deviation was characterized by strong NMR signals, predominantly in the aromatic region of the NMR spectrum, which could not be identified as known metabolites. Whereas the 10 infants with outlier samples had a somewhat lower gestational age than the others, there were no significant differences with respect to the nutritional intervention, SGA status, sex or infections or any other of the clinical parameters (data not shown).

The PCA loadings (not shown) revealed that the first principal component (PC1, the *x*-axis) of Figure 3 corresponded to increasing levels of citrate, betaine, glycine and hydroxyproline from right to left, along with decreasing unidentified spectral signals at 0.57 and 5.50 ppm. The second (PC2, the *y*-axis) and the third principal component (not shown) were dominated by the unidentified signals of the outlier samples mentioned above.

The metabolites were studied in relation to changes over time in a linear mixed model for repeated measures (Table 3). There was no significant interaction between intervention group and time for any of the metabolites, thus interaction terms were not included in the final models. There was no significant effect of the intervention, but for most of the metabolites, there was a significant effect of time. The levels of amino acids and many other metabolites increased between weeks 1 and 3, whereas gluconate and two strong, unidentified signals at positions 0.57 and 5.50 ppm disappeared. The results were similar irrespective of whether the outliers were kept or removed in the analyses (data not shown).

**Table 3:** Mixed model and change of mean metabolite levels between sampling weeks 1, 3, 5 and 7 (*n* = 48).

Metabolite	Weeks 1→7 Mixed Model			Weeks 1→3	Weeks 3→5	Weeks 5→7
				(*n* = 35 Pairs)	(*n* = 28 Pairs)	(*n* = 19 Pairs)
	*p* Interaction	*p* Diet	*p* Time	FC	FC	FC
Total Integral				−1.2	−1.2	1.2
1-Methylnicotinamide	0.21	0.24	0.02	1.2	1.0	−1.1
2-Oxoglutarate	0.05	0.51	<0.001	4.1	1.4	1.2
4-Hydroxyphenylacetate	0.43	0.11	<0.001	5.1	−1.1	−1.3
Betaine	0.96	0.16	<0.001	1.5	1.2	1.3
Citrate	0.80	0.26	<0.001	5.5	1.9	1.5
Creatinine	0.79	0.52	0.01	1.0	1.1	1.2
Dimethylamine	0.87	0.39	0.003	1.1	1.1	1.0
Fumarate	0.06	0.29	<0.001	3.2	1.7	1.0
Gluconate	-	-	-	x	-	-
Glycine	0.62	0.05	<0.001	1.6	1.2	−1.1
Hipurate	0.56	0.10	0.31	−1.2	1.6	1.0
Histidine	0.70	0.31	<0.001	1.2	1.6	−1.2
myo-Inositol	0.60	0.28	0.08	1.3	1.0	−1.2
*N*,*N*-Dimethylglycine	0.23	0.62	<0.001	1.3	1.2	1.3
Succinate	0.42	0.67	<0.001	5.0	1.4	1.0
Sucrose/Maltose	0.12	0.51	<0.001	−8.4	1.1	−1.1
Sugar doublets, 5.23 ppm	0.30	0.08	0.03	1.0	−1.2	−1.2
Threonine	0.58	0.34	<0.001	2.0	1.0	−1.7
*trans*-4-Hydroxy-l-proline	0.26	0.16	<0.001	3.1	1.3	1.0
Tyrosine	0.33	0.37	<0.001	3.7	1.0	−1.4
Unknown, 0.57 ppm	-	-	-	x	-	-
Unknown, 5.50 ppm	-	-	-	x	-	-
Unknown, 7.68 ppm	0.60	0.48	<0.001	1.2	1.1	1.1
Unknown, 7.76 ppm	0.43	0.9	<0.001	1.6	1.4	1.3

Linear mixed model for intervention (diet) and week of life (time) with adjustment for gestational age (GA) at birth and small for gestational age (SGA) status was used. Statistical significance was assumed for *p* < 0.002. Increase or decrease of log-transformed pseudo-concentrations, presented as fold-change (FC; ratios below 1 are presented as −1/ratio). The FC is based on available paired urine samples from the same child at the respective weeks of age. Metabolites marked “x” disappeared entirely; FC is therefore not applicable. Histidine is an uncertain assignment, based on a narrow doublet at 7.9–8.0 ppm. Total integral of the urine spectra was determined early relative to the added internal standard trimethylsilylpropionate-d4 (TSP) and then used to normalize all specified compounds.

Multivariate PLS regression analyses were carried out between the NMR spectra and clinical variables (Table 4). The nutritional intervention, presence of infections, and infants’ sex did not influence the urine spectra, whereas SGA status did show an effect on the metabolite profiles: The PLS model of the infants’ SGA status based on all spectra except those of the outliers reached a Q^2^/R^2^ ratio of 0.40. This increased to 0.53 by focusing on spectra from the first week of life, whereas spectra of the urine samples at 36 weeks PMA could not be linked to SGA status. Inclusion of the outliers in the analysis did not change these results (data not shown). PLS regression analysis also indicated that PMA as well as chronological age were associated with the urine spectra.

**Table 4:** Partial least squares regression of variables to sample spectra.

Variable	Samples ^a^	A ^b^	Q^2^	R^2^	Q^2^/R^2^
Intervention	all		-	-	-
	first		-	-	-
	36 weeks PMA		-	-	-
SGA status	all	3	34%	84%	0.40
	first	1	27%	50%	0.53
	36 weeks PMA		-	-	-
Infections	all		-	-	-
	first		-	-	-
	36 weeks PMA		-	-	-
Age (since birth)	all	2	41%	81%	0.51
	36 weeks PMA		-	-	-
Age (PMA)	all	3	67%	89%	0.75
	first	1	54%	70%	0.76
Sex	all		-	-	-
	first		-	-	-
	36 weeks PMA		-	-	-

R^2^: Endpoint variation contained in regression model. Q^2^: Variation reproduced in cross-validation. Higher numbers, or at least high Q^2^/R^2^ ratios mean reliable models. ^a^ Samples: All except outliers, first urine sampled from subject, and sample from 36 weeks PMA; ^b^ Number of PLS components resulting in best (highest) Q^2^/R^2^ ratio; results with Q^2^/R^2^ ratio below 0.3 not shown.

Observations linking the first-week urine samples to SGA status as well as PMA were also studied by linear regression analyses for selected metabolites (Table 5). In simple linear regression analyses, SGA status was associated with increased levels of glycine, histidine and threonine (8 × 10^−4^ ≤ *p* ≤ 0.003), as well as creatinine, succinate and *trans*-4-hydroxy-l-proline (hydroxyproline) (0.016 ≤ *p* ≤ 0.039). PMA was associated with a broader range of metabolites (3 × 10^−7^ ≤ *p* ≤ 0.040, for all variables in Table 5). The SGA infants had a higher mean gestational age at birth than AGA infants (29.9 *vs.* 27.5 weeks, *p* = 0.003). When adjusting for PMA in the multiple linear regression analyses, there is an indication that SGA was associated with glycine (*p* = 0.027) and threonine (*p* = 0.033), although not significant at the adjusted significance level.

**Table 5:** Linear regression results for selected metabolites at week 1 with respect to SGA status and PMA.

Metabolite ^a^	SGA Alone ^b^		PMA Alone ^c^		Mutually Adjusted ^d^			
(Week 1)	FC	*p*	FC	*p*	FC (SGA)	*p* (SGA)	FC (PMA)	*p* (PMA)
2-Oxoglutarate	1.6	0.15	1.3	4 × 10^−^^4^	−1.1	0.81	1.3	0.001
Betaine	1.4	0.057	1.1	9 × 10^−^^5^	1.0	0.95	1.1	5 × 10^−^^4^
Citrate	1.9	0.12	1.3	0.001	−1.1	0.87	1.3	0.004
Creatinine	1.8	0.018	1.2	3 × 10^−^^7^	1.0	0.86	1.2	6 × 10^−^^6^
Dimethylamine	1.5	0.036	1.2	2 × 10^−^^4^	1.1	0.69	1.1	0.001
Formate	1.6	0.083	1.2	0.005	1.1	0.66	1.1	0.022
Glycine	1.8	2 × 10^−^^4^	1.1	7 × 10^−^^5^	1.4	0.027	1.1	0.005
Histidine	2.0	8 × 10^−4^	1.2	9 × 10^−^^6^	1.4	0.091	1.2	6 × 10^−^^4^
myo-Inositol	1.5	0.062	1.1	0.001	1.4	0.73	1.1	0.006
*N*,*N*-Dimethylglycine	1.0	0.93	1.2	0.020	−1.6	0.18	1.2	0.008
Succinate	2.1	0.039	1.3	3 × 10^−^^4^	1.2	0.63	1.3	0.003
Sugar doublets, 5.23 ppm	−1.3	0.11	−1.1	0.002	−1.1	0.66	−1.1	0.012
Threonine	1.8	0.003	1.1	0.040	1.6	0.033	1.0	0.37
*trans*-4-Hydroxy-l-proline	1.9	0.016	1.3	5 × 10^−^^7^	1.1	0.81	1.3	1 × 10^−^^5^

Histidine is an uncertain assignment, based on a narrow doublet at 7.9–8.0 ppm. Significance assumed for *p* < 0.002. ^a^ Only selected contributions are shown; ^b^ Simple linear regression analyses of log-transformed pseudo-concentrations and SGA status; results are presented as fold-change (FC), e.g., glycine levels were 1.8-fold higher in the SGA group and 1.1-fold higher for each week’s difference in PMA at week 1 (ratios below 1 are presented as −1/ratio); ^c^ Corresponding analyses for PMA; FC is per one week difference in PMA; ^d^ Multiple linear regression including both SGA status and PMA.

**Figure 4:**
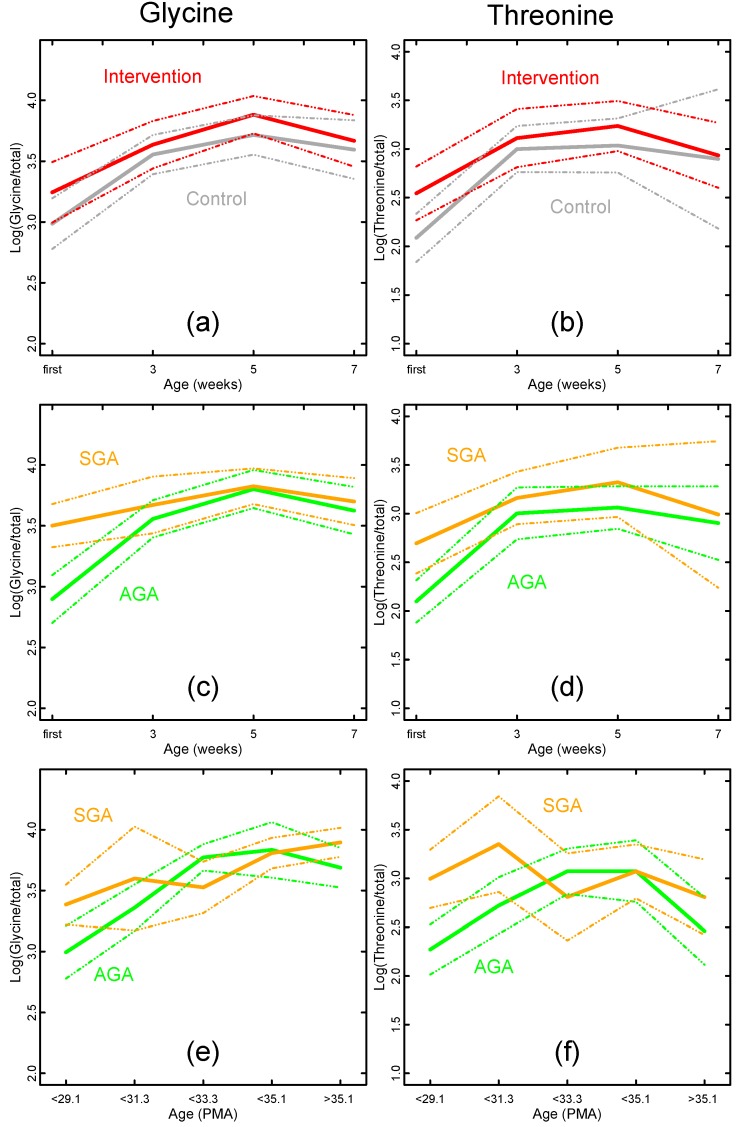
Temporal development of glycine and threonine log-pseudo-concentrations (means and 95% CIs) related to nutritional intervention, SGA status and age. (**a**) Glycine levels by nutritional intervention (intervention red, control gray); (**c**) Glycine levels by SGA status (SGA orange, AGA green) for samples from weeks 1, 3, 5 and 7; (**e**) As above, but samples selected by PMA instead of weeks of life; (**b**, **d**, **f**) Corresponding figures for threonine.

The previous considerations are summarized for the urinary metabolites glycine and threonine in Figure 4. There were similar levels of glycine and threonine in the intervention and control group (Figure 4a,b). Glycine and threonine levels appeared to differ between SGA and AGA children in the first week of life, but not at later time points (Figure 4c,d). The same applied when the infants’ age was defined as PMA instead of chronological age (Figure 4e,f).

Finally, the metabolite pseudo-concentrations were examined with respect to growth velocity from birth to four weeks of life [9]. In the initial linear regression models, first week glycine and threonine levels and third week glycine and hydroxyproline levels correlated positively with growth velocity. However, when adjusting for PMA in the models, these relations disappeared.

## 4. Discussion

Due to proposed risks of overfeeding, we investigated the impact of enhanced nutrition on the urinary excretion profile during the first weeks of life in premature infants. We did not observe significantly different metabolic trajectories between the intervention group receiving nutritional support in the upper range of recent recommendations as compared to the infants on standard nutrient supply; neither in the first-week urine profiles nor in the change over time. Furthermore, infants in the intervention group exhibited better overall growth [9]. Together, this suggests that premature infants handle enhanced nutrient supply similarly to a standard diet because the urinary profiles in the intervention group did not indicate an overload of the renal function as compared to the controls.

Our study also revealed that all infants exhibited substantial changes in their urinary profiles during the early postnatal period (Figure 3, Table 3), and these changes correlated with gestational age at birth and with chronological age (Table 3 and Table 5). The correlation between PMA and urinary metabolite profiles has been reported previously [17,31,32], and may reflect the degree of organ development and metabolic maturity. Between the first and the third week of life the glucogenic amino acids glycine, threonine, hydroxyproline and tyrosine increased along with metabolites of the tricarboxylic acid cycle like 2-oxoglutarate, citrate, fumarate and succinate. In most mammals, the prenatal-postnatal transition is accompanied by important adaptations in carbohydrate metabolism due to the abrupt change from the placental supply of nutrients to a cyclic supply of nutrients via the breast milk [7]. In rodents this period is characterized by the appearance of gluconeogenic enzymes to maintain glucose homeostasis in the newborn [33]. Thus, the metabolite changes observed in our premature infants might reflect similar metabolic adaptations. The presence of metabolites linked to the tricarboxylic acid cycle may be due to the high metabolic turnover in premature infants. The tricarboxylic acid cycle is important in energy metabolism, providing intermediates for the synthesis of glucose and some amino acids [34].

Hydroxyproline showed a threefold increase in concentration during the initial postnatal period. Urinary hydroxyproline reflects collagen metabolism and is considered a marker of infant growth [35,36]. Although we observed a positive correlation between third week hydroxyproline levels and growth velocity, this correlation disappeared when PMA was introduced as a covariate, suggesting that urinary hydroxyproline is closely related to PMA.

In parallel with the increase of the other metabolites during the first month of life, gluconate and two unidentified metabolites with an NMR signals at 5.50 and 0.57 ppm disappeared. The latter unidentified metabolite has also been observed in the urine of pregnant women [26,37]. Its disappearance shortly after birth suggests that this substance was transferred from the mother to the infant and may reflect a sulfate- or glucuronide-conjugate of pregnanediol or estrogen [26].

Most SGA infants have been exposed to a limited nutrient supply during fetal life, which may cause irreversible metabolic changes (fetal programming). Subsequent catch-up growth, both in early infancy and in childhood, is also associated with later obesity and cardiovascular disease risk [4,5,8]. The so-called mismatch hypothesis proposes that an obesogenic childhood environment increases later cardiovascular disease risk, whereas the postnatal programming or postnatal growth acceleration hypothesis links rapid weight gain in early infancy to later cardiovascular disease risk. In a recent review [3], growth during late infancy and childhood appeared to be the major determinant of later metabolic and cardiovascular disease risk, and not the early postnatal growth. It has also been shown that early postnatal growth has a significant impact on later neurodevelopment [3]. Both these findings support the aggressive nutritional approach in our intervention. We studied metabolic differences between SGA and AGA infants at birth and over time, and identified glycine and threonine as potential biomarkers of an altered metabolic phenotype.

Glycine has been linked to nutrient restriction of pregnant baboons, where the fetal plasma levels more than doubled compared to control fetuses [38]. A similar increase in fetal glycine levels has been observed in human SGA fetuses [39,40]. Dessi *et al.*, profiled newborn urines one and four days after birth and reported that in addition to the glycine and threonine pathway, prenatal growth restriction also affected metabolic pathways involving hydroxyproline, creatinine and myo-inositol [19]. They interpreted these metabolites as potential early markers of the metabolic syndrome. In line with their study, we found similar differences in the first-week urine profiles between our SGA and AGA infants, although after adjusting for PMA, only threonine and glycine remained independently elevated in the SGA infants. Glycine and threonine are glucogenic amino acids, which may be converted to pyruvate during protein metabolism. Increased levels of plasma glycine may be caused by reduced amino acid oxidation or reduced gluconeogenesis as a strategy to conserve amino acids [38]. We observed that glycine and threonine were linked to SGA status in the first urine sample, but we were unable to detect a persistent difference during the course of time. Although our study did not exhibit similar results for all metabolites as compared to the study by Dessi *et al.* [19], and the elevation of glycine and threonine levels were insignificant after Bonferroni adjustment, it still highlights glycine, threonine and to some extent hydroxyproline as important targets for future research.

Our study has several limitations. In our original intervention trial [10], we planned to recruit 240 infants. The early termination due to increased occurrence of septicemia in the intervention group resulted in a reduced number of infants in our present study. In spite of the fact that the intervention group had a significantly lower mean birth weight and a higher proportion of SGA infants than the control group, we observed increased whole body growth [9], improved white matter maturation and motion perception in the intervention group (manuscript submitted). Moreover, we did not find any significant differences between the metabolic trajectories with regard to the two different diets. The occurrence of septicemia and electrolyte deviations did not seem to influence the urinary metabolite profiles, but the lack of significant differences must be interpreted with caution in view of the relatively small study sample. Similar electrolyte disturbances have been reported in other studies with early and enhanced nutrition to premature infants during the first week of life [41,42,43,44,45,46]. Thus, a difference in metabolic profile would probably occur during the early postnatal stay, and it raises the question as to whether we would have been able to identify an effect with a more frequent first week monitoring of the urines in our premature infants.

Multiple hypothesis testing was performed using Bonferroni correction. Although samples from such vulnerable patients are challenging to come by, the randomized design of the current trial as well as the strict adherence to the nutritional protocol reduced the number of confounding factors.

## 5. Conclusions

The urinary metabolite profiles were unaltered by the enhanced postnatal nutrition, suggesting that supply in the upper range of current recommendations did not overload renal function. Our data show that both gestational age at birth, *i.e.*, degree of maturation, and postnatal physiological adaptations, may influence metabolism in premature infants during the neonatal period. Several of the first-week urinary metabolites were associated to SGA status and postnatal growth and might be markers for long-term health outcomes.
